# Improved COVID-19 Outcomes following Statin Therapy: An Updated Systematic Review and Meta-analysis

**DOI:** 10.1155/2021/1901772

**Published:** 2021-09-23

**Authors:** Amir Vahedian-Azimi, Seyede Momeneh Mohammadi, Maciej Banach, Farshad Heidari Beni, Paul C. Guest, Khalid Al-Rasadi, Tannaz Jamialahmadi, Amirhossein Sahebkar

**Affiliations:** ^1^Student Research Committee, Hamadan University of Medical Sciences, Hamadan, Iran; ^2^Department of Anatomical Sciences, Faculty of Medicine, Zanjan University of Medical Sciences, Zanjan, Iran; ^3^Department of Hypertension, Medical University of Lodz (MUL), Poland; ^4^Cardiovascular Research Centre, University of Zielona-Gora, Zielona-Gora, Poland; ^5^Nursing Care Research Center (NCRC), School of Nursing and Midwifery, Iran University of Medical Sciences, Tehran, Iran; ^6^Laboratory of Neuroproteomics, Department of Biochemistry and Tissue Biology, Institute of Biology, University of Campinas (UNICAMP), Campinas, Brazil; ^7^Medical Research Centre, Sultan Qaboos University, Muscat, Oman; ^8^Department of Food Science and Technology, Quchan Branch, Islamic Azad University, Quchan, Iran; ^9^Department of Nutrition, Faculty of Medicine, Mashhad University of Medical Sciences, Mashhad, Iran; ^10^Applied Biomedical Research Center, Mashhad University of Medical Sciences, Mashhad, Iran; ^11^Biotechnology Research Center, Pharmaceutical Technology Institute, Mashhad University of Medical Sciences, Mashhad, Iran; ^12^School of Pharmacy, Mashhad University of Medical Sciences, Mashhad, Iran

## Abstract

**Background:**

Although vaccine rollout for COVID-19 has been effective in some countries, there is still an urgent need to reduce disease transmission and severity. We recently carried out a meta-analysis and found that pre- and in-hospital use of statins may improve COVID-19 mortality outcomes. Here, we provide an updated meta-analysis in an attempt to validate these results and increase the statistical power of these potentially important findings.

**Methods:**

The meta-analysis investigated the effect of observational and randomized clinical studies on intensive care unit (ICU) admission, tracheal intubation, and death outcomes in COVID-19 cases involving statin treatment, by searching the scientific literature up to April 23, 2021. Statistical analysis and random effect modeling were performed to assess the combined effects of the updated and previous findings on the outcome measures. *Findings*. The updated literature search led to the identification of 23 additional studies on statin use in COVID-19 patients. Analysis of the combined studies (*n* = 47; 3,238,508 subjects) showed no significant effect of statin treatment on ICU admission and all-cause mortality but a significant reduction in tracheal intubation (OR = 0.73, 95% CI: 0.54-0.99, *p* = 0.04, *n* = 10 studies). The further analysis showed that death outcomes were significantly reduced in the patients who received statins during hospitalization (OR = 0.54, 95% CI: 0.50-0.58, *p* < 0.001, *n* = 7 studies), with no such effect of statin therapy before hospital admission (OR = 1.06, 95% CI = 0.82-1.37, *p* = 0.670, *n* = 29 studies).

**Conclusion:**

Taken together, this updated meta-analysis extends and confirms the findings of our previous study, suggesting that in-hospital statin use leads to significant reduction of all-cause mortality in COVID-19 cases. Considering these results, statin therapy during hospitalization, while indicated, should be recommended.

## 1. Introduction

As of May 1, 2021, 152,038,419 people have been infected by the SARS-CoV-2 virus, the cause of Coronavirus Disease 2019 (COVID-19) [1, 2]. This translates to nearly 2% of the world population and accounts for a doubling in the number of cases over the last 6 months [3]. The number of people who have died in association with a COVID-19 diagnosis has now reached 3,194,337, which translates to a death rate that has held steady over the last 6 months at 2.1% of the cases. However, since December 2020, we have seen the rollout and administration of multiple vaccines against COVID-19 disease, due to an unprecedented and coordinated effort across the world. Although some countries with advanced vaccination programs have seen a reduction in COVID-19 case numbers, there is still an urgency to control disease spread and reduce its severity worldwide.

While waiting for increased vaccinations across the globe, one way of achieving this is through repurposing existing therapeutics. We recently carried out a meta-analysis, which identified significant reductions in intensive care unit (ICU) admission and death outcomes in COVID-19 patients taking statins [4]. Most importantly, this analysis also found that mortality was reduced most profoundly in those patients who were administered statins in-hospital (by 60%), compared to those who were already taking statins prior to hospital admission (by 23%). If confirmed, this would represent an important step forward in the treatment of COVID-19 disease severity. However, this latter finding was accounted for by only three studies with significant heterogeneity between them [4]. In addition, a recent meta-analysis by Hariyanto and Kurniawan [5] indicated that statin use has nothing to do with the composite adverse outcomes of COVID-19, including the risk of mortality. However, the study showed that despite the presence of COVID-19 infection, patients with dyslipidemia should continue to take statins as this is beneficial for cardiovascular outcomes.

Here, we provide an updated meta-analysis to further compare statin use on ICU admission, tracheal intubation, and death outcomes in COVID-19 patients. It was of particular interest to compare in-hospital vs. prehospital statin treatment on these outcomes.

## 2. Methods

### 2.1. Search Strategy

This meta-analysis was performed according to PRISMA guidelines. The searches were reconducted using Web of Science, PubMed, Scopus, and ProQuest databases for targeted articles up to April 23, 2021 (previous searches had been performed up to November 2, 2020). The population, intervention, comparison, and outcome (PICO) criteria were, respectively, patients infected with qPCR-confirmed SARS-CoV-2, statin therapy, SARS-CoV-2 patients who were not treated with statins, and intensive care unit (ICU) admission, tracheal intubation, and mortality.

The main aim was to further elucidate if statin therapy is associated with the improvement of outcomes in COVID-19 patients. The keywords were chosen as described previously to account for the various names of SARS-CoV-2 and statins [4]. For comprehensive screening of target articles, we first carried out searches without consideration of specific outcomes. Next, we identified three outcomes (ICU admission, tracheal intubation, and mortality) that could be used in a well-powered meta-analysis.

### 2.2. Eligibility Criteria

The inclusion criteria were (1) observational studies and randomized clinical trials testing the effect of statins on COVID-19 and (2) studies including ICU admission, tracheal intubation, and mortality outcomes. Articles were excluded if they were (1) clinical case reports, literature reviews, and preclinical investigations and (2) studies which did not incorporate statin nonusers as controls.

### 2.3. Quality Assessment

Assessment of study quality was performed separately by two authors (FHB and AVA), applying the Newcastle-Ottawa Scale (NOS) for cohort studies, and disagreements were resolved as above. The assessment categories were (1) selection of study groups, (2) comparability of groups, and (3) ascertainment of either the exposure (case-control studies) or outcome (cohort studies) of interest. These were rated from 0 to 3 stars as an indication of quality. This translated to a total of 0 to 9 stars per article.

### 2.4. Statistical Analysis

The analyses were conducted as described previously [4]. Briefly, data extraction for the main outcomes was performed, and random effect meta-analysis was conducted, by applying the restricted maximum likelihood method [6], to account for unknown, unregistered, or unpublished studies. Heterogeneity between studies was determined using the Cochran *Q* test, tau-squared (*τ*^2^), *H*-squared (*H*^2^), and *I*-squared (*I*^2^) statistics. Significant results and *I*^2^ values higher than 75% were considered heterogeneous while *H*^2^ = 1 represented perfect homogeneity [7]. Publication biases were displayed using funnel plots, and regression-based Egger's [8] and nonparametric rank correlation-based Begg's [9] tests were applied as a measure of small-study effects. A nonparametric “trim and fill” method was used to account for publication bias, and modified effect sizes were estimated. Common effect sizes were displayed using an odds ratio (OR) with 95% confidence interval (CI) for the outcomes, and forest plots were used to illustrate the significance of the results. Subgroup analyses were performed for those studies reporting in- or prehospital use of statins.

## 3. Results

### 3.1. Literature Search

Supplementary Figure 1 shows the flowchart of the study selection process. A total of 1,234 records were initially searched from PubMed (*n* = 319), Scopus (*n* = 206), Web of Science (*n* = 652), and ProQuest (*n* = 49), and 8 studies were identified through other sources. The full list of records was reviewed with 144 duplicate studies omitted from the study, leaving 1,090 records. Following this, articles were screened by titles and abstracts, and the full texts of the remaining 323 studies were evaluated for eligibility. This left 71 studies for the final stringent screen. Finally, 47 studies were included, which met the eligibility criteria. Odds ratios (ORs) were extracted to evaluate the effect of statin use in patients with COVID-19 on ICU admission (*n* = 17), tracheal intubation (*n* = 10), and death (*n* = 41). The general characteristics of included studies are given in Table 1. In addition, quality assessment of studies was done by the Newcastle-Ottawa scale (Supplementary Table 1).

### 3.2. ICU Admission

**Figure 1:**
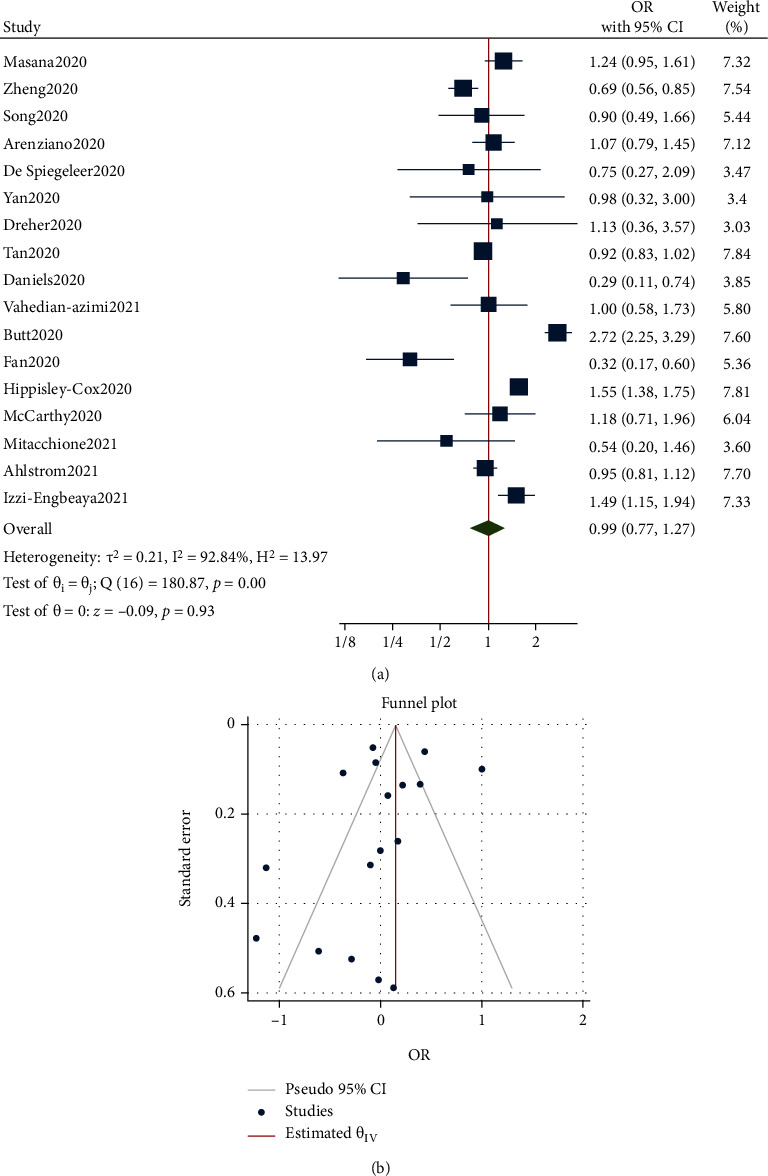
(a) Forest plot showing the risk of ICU admission between statin and nonstatin users in patients with COVID-19. (b) Funnel plot showing publication bias on ICU admission risk between statin and nonstatin users in patients with COVID-19.

As shown in Figure 1(a), the risk of ICU admission between statin and nonstatin users in patients with COVID-19 was not significant. The OR from 17 studies was 0.99 (95% CI: 0.77-1.27, *p* = 0.930) with significant heterogeneity between studies (*τ*^2^ = 0.21, *I*^2^ = 92.84%, *H*^2^ = 13.97, *Q*_(df = 16)_ = 180.87, *p* < 0.001). Assessment for bias by Egger's (*p* = 0.066) and Begg's (*p* = 0.295) tests did not find significant small-study effects, and visual analysis of the funnel plot showed some publication bias effects (Figure 1(b)).

### 3.3. Tracheal Intubation

**Figure 2:**
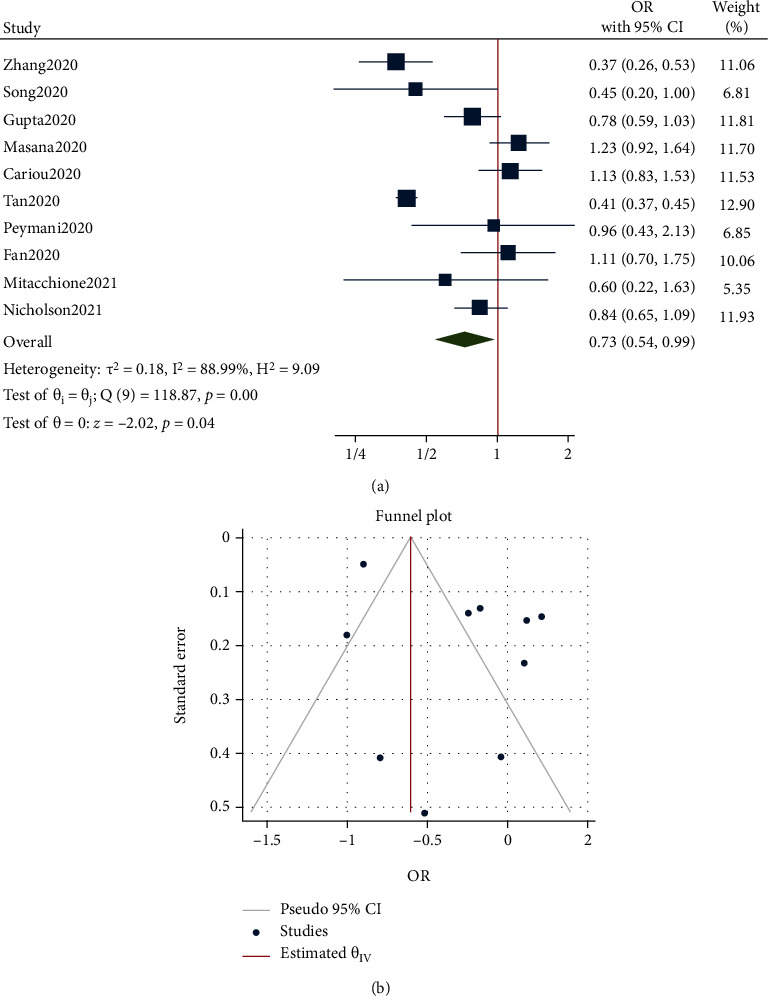
(a) Forest plot showing the risk of tracheal intubation between statin and nonstatin users in patients with COVID-19. (b) Funnel plot showing publication bias on tracheal intubation risk between statin and nonstatin users in patients with COVID-19.

As shown in Figure 2(a), the risk of tracheal intubation between statin and nonstatin users in patients with COVID-19 was significantly different. The risk of tracheal intubation in patients with COVID-19 who used statins was significantly reduced by 27% compared with those who did not take statins. The OR from 10 studies was 0.73 (95% CI: 0.54-0.99, *p* = 0.04), with significant heterogeneity between studies (*τ*^2^ = 0.18, *I*^2^ = 88.99%, *H*^2^ = 9.09, *Q*_(df = 9)_ = 118.87, *p* < 0.001). Small-study effects were not significant as shown by Egger's (*p* = 0.993) and Begg's (*p* = 0.236) tests, and the funnel plot suggested no publication bias (Figure 2(b)). Thus, the results were not extended to account for publication bias.

### 3.4. Death

**Figure 3:**
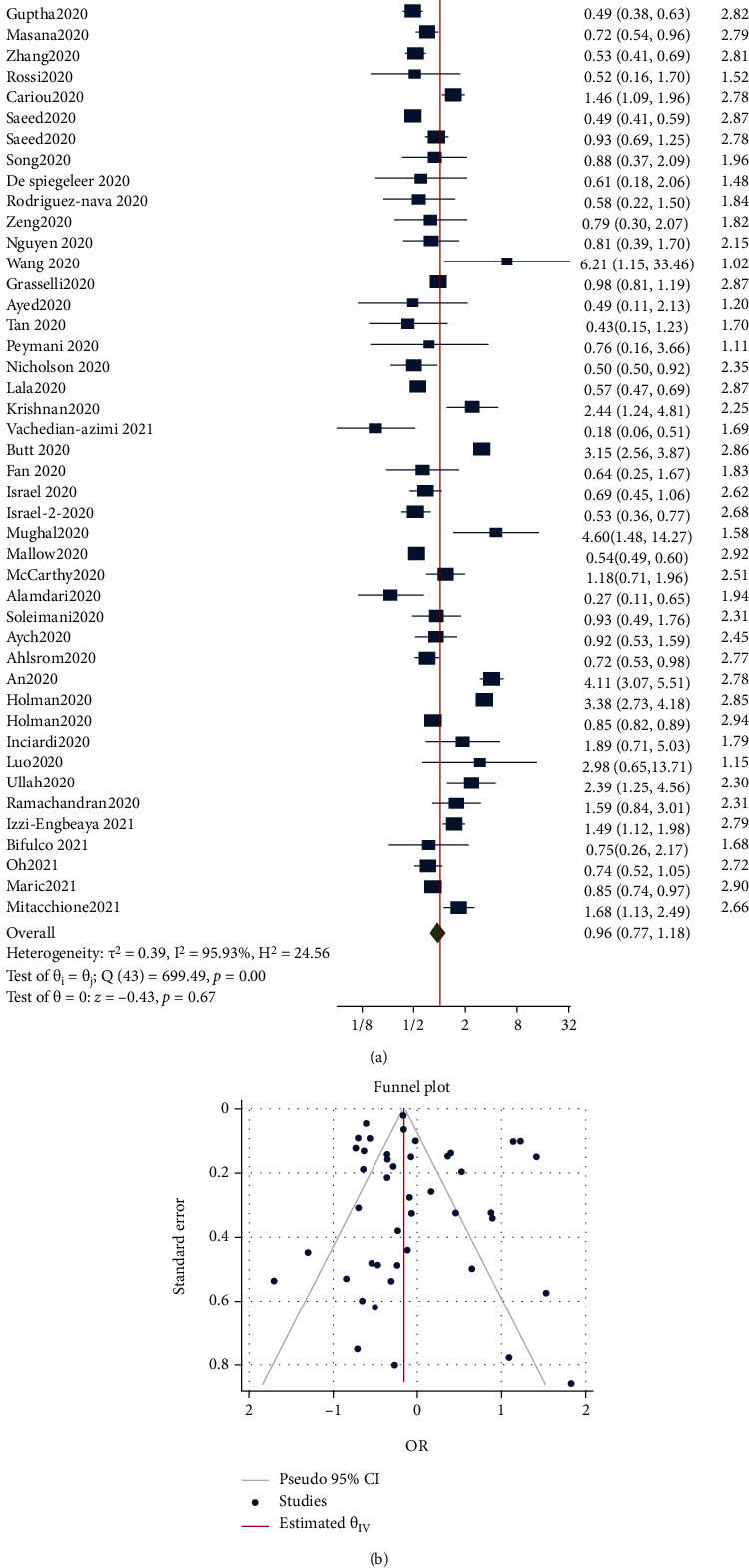
(a) Forest plot showing the risk of mortality between statin and nonstatin users in patients with COVID-19. (b) Funnel plot showing publication bias on mortality risk between statin and nonstatin users in patients with COVID-19.

As shown in Figure 3(a), the risk of mortality between statin and nonstatin users in patients with COVID-19 was not significant. The OR from the 41 studies which determined the effect of statins on mortality was 0.96 (95% CI: 0.77-1.18, *p* = 0.67), with significant heterogeneity between studies (*τ*^2^ = 0.39, *I*^2^ = 95.93%, *H*^2^ = 24.56, *Q*_(df = 43)_ = 699.49, *p* < 0.001). Assessment for bias by Egger's (*p* = 0.953) and Begg's (*p* = 0.551) tests showed no significant small-study effects, and visual inspection of the funnel plot suggested no publication bias (Figure 3(b)). When the analysis was restricted to studies in populations with cardiovascular disease (*n* = 3) and diabetes (*n* = 4), total death was found to be reduced in the former (OR = 0.62 (95% CI: 0.45-0.85, *p* < 0.001)) but not the latter (OR = 1.06 (95% CI: 0.46-2.41, *p* = 0.890)).

**Figure 4:**
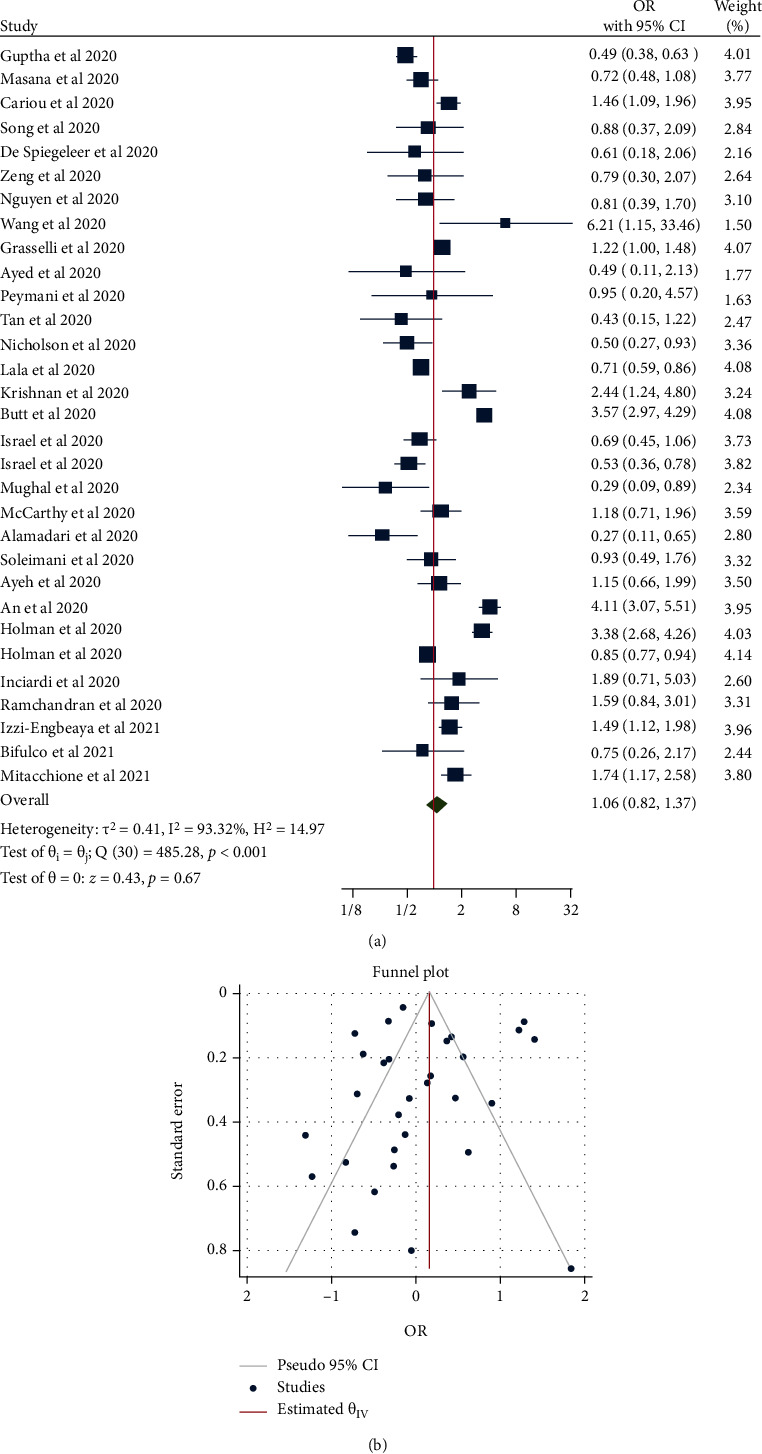
(a) Forest plot showing the risk of mortality in patients with COVID-19 who used statins prehospital compared with those who did not take statins. (b) Funnel plot showing publication bias on mortality risk in patients with COVID-19 who used statins prehospital compared with those who did not take statins.

The risk of mortality in patients with COVID-19 who used statins before hospital admission was not significantly different from those who did not take statins (OR = 1.06, 95% CI = 0.82-1.37, *p* = 0.670, 29 studies) but with significant heterogeneity between studies (*τ*^2^ = 0.41, *I*^2^ = 93.32%, *H*^2^ = 14.97, *Q*_(df = 30)_ = 485.28, *p* < 0.001) (Figure 4(a)). Analysis using Egger's (*p* = 0.167) and Begg's (*p* = 0.316) tests found no significant small-study effects, and the funnel plot showed no publication bias (Figure 4(b)). In the subgroup of studies conducted in populations with cardiovascular disease (*n* = 2; OR = 0.66, 95% CI = 0.43-1.02, *p* = 0.060) or diabetes (*n* = 3; OR = 1.12, 95% CI = 0.36-3.44, *p* = 0.840), there was no significant effect of prehospital statin use on mortality.

**Figure 5:**
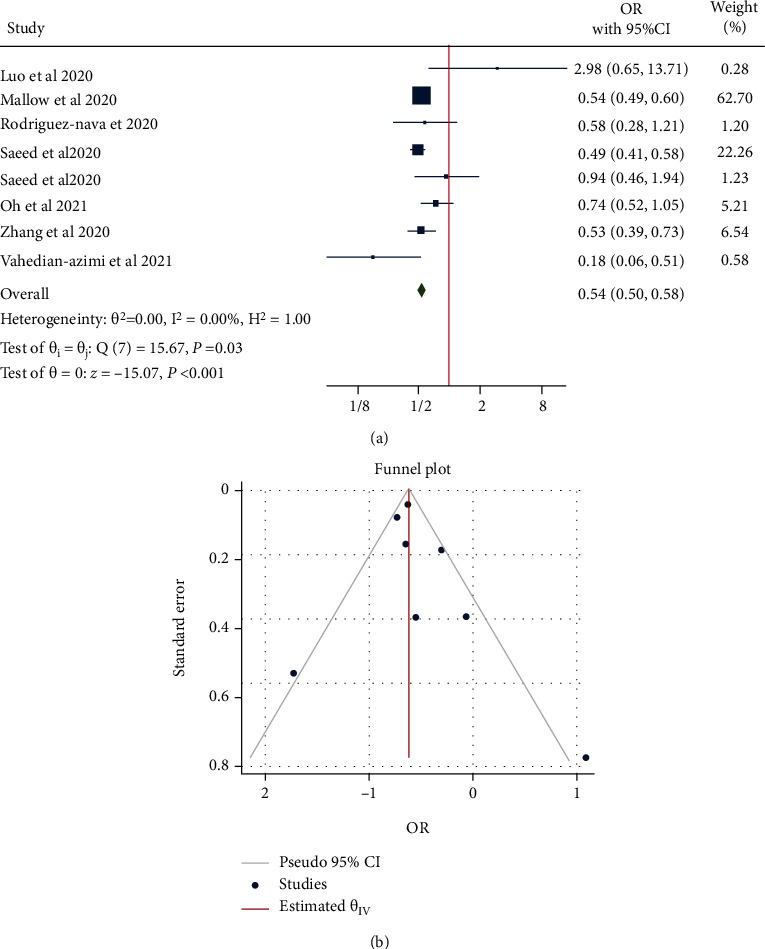
(a) Forest plot showing the risk of mortality in patients with COVID-19 who used statins in-hospital compared with those who did not take statins. (b) Funnel plot showing publication bias on mortality risk in patients with COVID-19 who used statins in-hospital compared with those who did not take statins.

We also analyzed mortality risk in COVID-19 patients who received statins only after hospital admission. This allowed analysis of a new total of 7 studies which found a significant reduction in mortality compared with those who did not take statins (OR = 0.54, 95% CI = 0.5-0.58, *p* < 0.001), with no significant heterogeneity between studies (*τ*^2^ = 0.00, *I*^2^ = 0.00%, *H*^2^ = 1, *Q*_(df = 30)_ = 15.67, *p* = 0.03) (Figure 5(a)). Egger's (*p* = 0.167) and Begg's (*p* = 0.316) testing showed no significant small-study effects, and the funnel plot suggested no publication bias (Figure 5(b)).

## 4. Discussion

Our updated meta-analysis found no significant reductions in ICU admission and mortality outcomes in COVID-19 patients who used statins, compared to those who were not on these drugs. Interestingly, a significant reduction of all-cause mortality with statins was observed in patients with cardiovascular disease; however, due to the limited number of studies included, this still needs to be confirmed. The subgroup analysis also showed that administration of statins during hospitalization was associated with a significant 46% reduction in mortality, in line with the findings of our previous study [10]. Conversely, we found that use of statins prior to admission had no significant effect on the mortality outcomes. What is additionally important, statin therapy also reduced tracheal intubation by 27%.

One possibility for these differences in mortality outcomes could be associated with the type of statin used across different studies. As the characteristic of the included studies were varied, this gives rise to bias which makes it difficult to draw firm conclusions. Expectedly, differential physiochemical characteristics of statins can affect the potency of their well-known pleiotropic actions [11–18]. For example, one study found that treatment with simvastatin or atorvastatin led to a reduction in mortality of COVID-19 patients, compared to cases given pravastatin or rosuvastatin [19]. In addition, the CORONADO study showed that treatment with statins was associated with increased mortality in COVID-19 patients with preexisting diabetes [20], although another study found that statin use reduced mortality in a similar patient group [21]. Again, this might have been due to the use of different statins as information regarding the statin type was not listed in the CORONADO study. Another possibility for the lack of effect of prehospital use of statins on mortality outcomes in COVID-19 patients could be due to the preexistence of diseases such as obesity, hypertension, cardiovascular disorders, and metabolic diseases, which are significant risk factors for severe outcomes [22–24]. This could be explained by the possibility that any potential benefit of statins could be nullified by the presence of comorbidities. Finally, the observed benefit in terms of reducing the incidence of tracheal intubation deserves further investigation. This benefit might imply that statin therapy is particularly beneficial in reducing the serious complications of COVID-19 like intubation which is closely related to death. This notion is in line with the observed mortality benefit in patients receiving statins during hospitalization.

The currently updated meta-analysis had several limitations. First and foremost, only associations are given since it was not possible to investigate a cause-and-effect relationship involving statin use. Secondly, we do not have data from the included studies on the preparations of statins that were used in COVID-19 patients, which is a reason we cannot make any conclusions whether there are differences in the outcomes between hydrophilic and lipophilic ones. Thirdly, potential effects of preexisting or postdiagnosis development of comorbidities such as acute respiratory distress, coagulation disorders, or insulin resistance cannot be excluded. Fourthly, the findings were not adjusted for other medication use, which may also have affected outcomes. Finally, although the number of studies that we identified which investigated in-hospital use of statins was more than doubled in this updated meta-analysis [10, 21, 25–29], this was still likely to have been statistically underpowered.

In conclusion, this updated meta-analysis further supports our previous finding that administration of statins during hospitalization is associated with reduced mortality of patients diagnosed with COVID-19 disease. Thus, further clinical studies are warranted to determine the timing of statin administration, recommended preparations, and doses, as well as potential effects of preexisting medical conditions and prescribed medications on clinical outcomes in COVID-19 patients. Most importantly, such studies will provide critical insights and outline strategic measures and patient-specific treatment approaches to successfully control the current devastating COVID-19 outbreak. It is hoped that such studies will help to pave the way for better preparedness in the likely event of future pandemics. However, more randomized clinical trial studies are needed to confirm these results.

## Figures and Tables

**Table 1:** Characteristics of included studies.

ICU admission								
Author	Statin		Sample	Setting	Study design	Result	Conclusion	Reference
	User	Nonuser						
Masana et al.	581	1576	Patients admitted to their hospitals because of SARS-CoV-2 infection	Members of the Lipids and Arteriosclerosis Units Net (XULA) of Catalonia (Spain)	Retrospective observational	N/A	N/A	[30]
	103 (17.7)	233 (14.8)						
Zhang et al.	1219	12762	Patients with COVID-19	Hubei Province, China	Retrospective	aHR: 0.69, CI: 0.56-0.85, *p* = 0.001	Cox model analysis showed statin use associated with lower prevalence ICU admission	[25]
	N/A	N/A						
Song et al.	123	126	Patients with COVID-19	“Lifespan” healthcare system hospitals	Retrospective cohort	OR: 0.90, CI: 0.49-1.67, *p* = 0.756	No significant associations between statin use and hospital death or ICU admission	[31]
	N/A	N/A						
Argenziano et al.	325	525	Patients with laboratory-confirmed COVID-19 infection	New York-Presbyterian/Columbia University Irving Medical Center, a quaternary care academic medical center	Retrospective case series	OR = 1.07, CI: 0.79-1.46	N/A	[32]
	93	143						
De Spiegeleer et al.	31	123	Residents at two elderly care homes with COVID-19 diagnosis	One of two Belgian nursing homes	Retrospective multicenter cohort	OR: 0.75, CI: 0.24-1.87	Statin use showed nonsignificant benefits	[33]
	6	31						
Yan et al.	N/A	N/A	Confirmed COVID-19 diagnosis	Hospitals in Zhejiang Province, China	Case-control	OR: 0.98, CI: 0.32-2.99, *p* = 0.973	N/A	[34]
	N/A	N/A						
Dreher et al.	18	32	COVID-19 patients with and without acute respiratory distress syndrome (ARDS)	Aachen University Hospital	Retrospective cohort	OR: 1.13, CI: 0.36-3.60	N/A	[35]
	9	15						
Tan et al.	40	509	717 patients admitted	Tertiary center in Singapore for COVID-19 infection	Retrospective cohort	ATET Coeff: − 0.12, CI: −0.23-0.01, *p* = 0.028	Statin use independently associated with lower requirement for ICU admission	[36]
	1	N/A						
Daniels et al.	46	124	Patients hospitalized for treatment of COVID-19	University of California San Diego Health (UCSDH), ascertained by data capture within system-wide electronic health record (EHR) system (Epic Systems, Verona, WI, USA)	Retrospective cohort	Adjusted OR: 0.29, CI: 0.11-0.71, *p* < 0.01	Inpatients hospitalized for COVID-19, use of statin medication prior to admission associated with reduced risk of severe disease	[37]
	20	70						
Vahedian-Azimi et al.	326	525	Positive for SARS-CoV-2	Baqiyatallah University of Medical Sciences	Prospective observational	OR: 1.00, CI: 0.58-1.74, *p* = 0.736	Statin use not associated with mortality	[10]
	39	243						
Butt et al.	843	3999	Danish citizens had a primary or secondary diagnosis code for COVID-19 infection	A Danish hospital, including inpatient, outpatient, and emergency department visits	Observational cohort study	HR 2.41 (95% CI 2.04 to 2.85)	Statin exposure was associated with a significantly higher risk of severe COVID-19 infection compared with no statin exposure)severe COVID-19 infection, defined as a hospital diagnosis of “COVID-19 severe acute respiratory syndrome” (ICD-10 code: B972A) or admission to an intensive care unit(	[38]
	204 (24.2%)	419 (10.5%)						
Fan et al.	250	1897	Patients with COVID-19	Zhongnan Hospital of Wuhan University and Leishenshan Hospital in Wuhan, China	Retrospective study	Adjusted HR, 0.319; 95% CI, 0.270–0.945; *p* = 0.032	The risk was lower for intensive care unit (ICU) care in the statin group vs. the nonstatin group	[39]
	N/A	N/A						
Hippisley-Cox et al.	5616	13870	Patients who had COVID-19 disease	General practices in England contributing to the QResearch database from which current data were available, England	Prospective cohort study	HR = 1.21 (1.02-1.43) OR = 1.55 (1.38-1.75)	For ICU admission, there was no significant associations with the statin	[40]
	487 (8.7%)	799 (5.8%)						
McCarthy et al.	107	140	Patients hospitalized with confirmed SARS-CoV-2 infection	Three Partners Healthcare hospitals (Massachusetts General Hospital, Brigham and Women's Hospital, and Newton-Wellesley Hospital)	Retrospective cohort study	Admitted to ICU or died OR: 1.18 (0.71-1.96)	N/A	[41]
	51	61						
Mitacchione et al.	179	663	Patients hospitalized for COVID-19	Hospitals include Luigi Sacco Hospital, Milan; Policlinico Umberto I Hospital, Rome; Spedali Civili Hospital, Brescia; Humanitas Gavazzeni Hospital; Bergamo, Italia	Observational multicenter study	*p* = 0.162	Our results did not confirm the supposed favorable effects of statin therapy on COVID-19 intensive care unit admission	[42]
	6 (3%)	40 (6%)						
Ahlström et al.	N/A	N/A	ICU COVID-19 patients	Sweden	Retrospective cohort study	OR = 0.95 (0.81-1.12) *p* = 0.53	We did not find a protective effect on ICU admission in statin-treated patients	[43]
	518	1466						
Izzi-Engbeaya et al.	N/A	N/A	Patients hospitalized with swab-positive COVID-19	ICHNT, which includes three hospitals admitting patients with COVID-19 (Charing Cross Hospital, Hammersmith Hospital, and St. Mary's Hospital), London	Retrospective cohort study	Primary outcome of death/ICU admission Estimate: −0.105 SE: 0.504 *p* = 0.835 OR = 1.49 (1.12-1.98)	N/A	[44]
	N/A	N/A						
Tracheal intubation								
Author	Statin		Sample	Setting	Study design	Result	Conclusion	
	User	Nonuser						
Zhang et al.	1219	12762	Patients with COVID-19	Hubei Province, China	Retrospective	aHR: 0.37, CI: 0.26-0.53, *p* < 0.001	Cox model analysis showed statin use associated with a lower prevalence of using mechanical ventilation	[25]
	N/A	N/A						
Song et al.	123	126	Patients with COVID-19	“Lifespan” healthcare system hospitals	Retrospective cohort	Statin use significantly associated with decreased risk for IMV OR: 0.45, CI: 0.20-0.99, *p* = 0.048	Data support continued use of statins in patients hospitalized with COVID-19 due to decreased risk for IMV	[31]
	N/A	N/A						
Gupta et al.	648	648	Positive for SARS-CoV-2	Columbia University Irving Medical Center (CUIMC) and Allen Hospital sites of the New York-Presbyterian Hospital (NYPH)	Retrospective	No significant difference in invasive mechanical ventilation	N/A	[45]
	130 (20.1%)	158 (24.4%)						
Masana et al.	581	1576	Patients admitted to hospitals due to SARS-CoV-2 infection	Members of the Lipids and Arteriosclerosis Units Net (XULA) of Catalonia (Spain)	Retrospective observational	N/A	N/A	[30]
	84 (14.46)	191 (12.12)						
Cariou et al.	1192	1257	Patients with diabetes admitted with COVID-19	68 French hospitals	Nationwide observational	OR: 1.13, CI: 0.83-1.53	Routine statin use not significantly associated with increased risk of tracheal intubation/mechanical ventilation	[46]
	19.2%	19.7%						
Tan et al.	40	509	Patients admitted for COVID-19	Tertiary center in Singapore for COVID-19 infection	Retrospective cohort	ATET Coeff: −0.08, CI: −0.19-0.02, *p* = 0.114	No significant differences in intubation	[36]
	1	N/A						
Peymani et al.	75	75	Hospitalized COVID-19 patients	Single tertiary hospital in Shiraz, Iran	Retrospective	OR: 0.96, CI: 0.61-2.99, *p* = 0.942	Nonsignificant association between statin use and reduction in mortality in COVID-19 patients	[47]
	N/A	N/A						
Fan et al.	250	1897	Patients with COVID-19	Zhongnan Hospital of Wuhan University and Leishenshan Hospital in Wuhan, China	Retrospective study	N/A	N/A	[39]
	26 (10.4%)	180 (9.4%)						
Mitacchione et al.	179	663	Patients hospitalized for COVID-19	Hospitals include Luigi Sacco Hospital, Milan; Policlinico Umberto I Hospital, Rome; Spedali Civili Hospital, Brescia; Humanitas Gavazzeni Hospital; Bergamo, Italia	Observational multicenter study	*p* = 0.258	Our results did not confirm the supposed favorable effects of statin therapy on COVID-19 mechanical ventilation	[42]
	6 (3%)	36 (5%)						
Nicholson et al.	511	531	Adult patients with laboratory-confirmed COVID-19 infection	Five hospitals in the Mass General Brigham healthcare system (Massachusetts General Hospital (MGH), Brigham and Women's Hospital (BWH), Newton Wellesley Hospital (NWH), Brigham and Women's Faulkner Hospital (BWFH), and North Shore Medical Center, NSMC) in Boston, USA	Retrospective cohort	OR = 0.84 (0.65–1.09), *p* = 0.182	N/A	[48]
	180	224						
Mortality								
Author	Statin		Sample	Setting	Study design	Result	Conclusion	Statin time
	User	Nonuser						
Gupta et al.	648	648	Positive for SARS-CoV-2	Columbia University Irving Medical Center (CUIMC) and Allen Hospital sites of the New York-Presbyterian Hospital (NYPH)	Retrospective	Univariate OR: 0.69, CI: 0.56-0.85. Multivariate adjusted OR: 0.49, CI: 0.38-0.63	Antecedent statin use associated with significantly lower rates of in-hospital mortality within 30 days	[45]
	112 (17.2%)	201 (31.0%)						
Masana et al.	581	581	Patients admitted to hospitals due to SARS-CoV-2 infection	Members of the Lipids and Arteriosclerosis Units Net (XULA) of Catalonia (Spain)	Retrospective observational	Significant difference in mortality rate between groups HR: 0.58, CI: 0.39-0.89, *p* = 0.01	A lower SARS-CoV-2 infection-related mortality observed in patients treated with statin therapy prior to hospitalization	[30]
	115 (19.79)	148 (25.40)						
Zhang et al.	1219	12762	Patients with COVID-19	Hubei Province, China	Retrospective	Individuals with statin therapy had a lower crude 28-day mortality (incidence rate ratios (IRR): 0.78, CI: 0.61–1.00, *p* = 0.046)	Statin use in hospitalized COVID-19 patients associated with lower risk of all-cause mortality and favorable recovery profile	[25]
	0.21%	0.27%						
Rossi et al.	42	29	Patients with preexisting chronic cardiovascular disease, with COVID-19	N/A	Observational	Mortality rates of patients taking statins were 21.4% (9/42) and 34.5% (10/29) in those not taking statins (*p* < 0.05)	Statin use significantly reduced risk of mortality in COVID-19 patients	[19]
	9 (21.4%)	10 (34.5%)						
Cariou et al.	1192	1257	Patients with diabetes admitted with COVID-19	68 French hospitals	Nationwide observational	Mortality rates significantly higher in statin users in 28 days (23.9% vs. 18.2%, *p* < 0.001). OR: 1.46, CI: 1.08-1.95	Routine statin treatment significantly associated with increased mortality in T2DM patients hospitalized for COVID-19	[46]
	23.9%	18.2%						
Saeed et al.	983	1283	Patients with diabetes mellitus hospitalized with COVID-19	Montefiore Medical Center, Bronx, New York	Observational retrospective	Patient with diabetes on statins had lower cumulative in-hospital mortality (24% vs. 39%, *p* < 0.01). HR: 0.51, CI: 0.43-0.61, *p* < 0.001	Statin use associated with reduced in-hospital mortality from COVID-19 in patients with diabetes	[21]
	24%	39%						
Saeed et al.	372	1614	Patients without diabetes mellitus hospitalized with COVID-19	Montefiore Medical Center in Bronx, New York	Observational retrospective	No difference noted in patients without diabetes (20% vs. 21%, *p* = 0.82)	Statin use associated with reduced in-hospital mortality from COVID-19 inpatients with diabetes	[21]
	20%	21%						
Song et al.	123	126	Patients with COVID-19	“Lifespan” healthcare system hospitals	Retrospective cohort	No significant associations between statin use and in-hospital death OR: 0.88, CI: 0.37-2.08, *p* = 0.781	No significant associations between statin use and hospital death	[31]
	N/A	N/A						
De Spiegeleer et al.	31	123	Residents at two elderly care homes with COVID-19 diagnosis	One of two Belgian nursing homes	Retrospective multicenter cohort	Considering death as serious outcome, the effect sizes, OR: 0.61, CI: 0.15-1.71, *p* = 0.380	Statins not statistically significantly associated with death from COVID-19 in elderly adults in nursing homes	[33]
	N/A	N/A						
Rodriguez-Nava et al.	47	40	Laboratory-confirmed COVID-19	Community hospital intensive care unit (ICU) located in Evanston, IL	Retrospective cohort	Multivariable Cox PH regression model showed atorvastatin nonusers had 73% chance of faster progression to death compared with users. HR: 0.38, CI: 0.18-0.77, *p* = 0.008	Slower progression to death associated with atorvastatin use in patients with COVID-19 admitted to ICU	[26]
	23 (49%)	25 (63%)						
Zenga et al.	38	993	COVID-19 inpatients	Tongji Hospital, Tongji Medical College of HUST (Wuhan, China)	Retrospective cohort	OR = 0.79, CI = 0.3-2.05	N/A	[49]
	5	160						
Nguyen et al.	90	266	African American population with COVID-19	University of Chicago Medical Center (UCMC), serving south metropolitan Chicago	Retrospective observational	OR = 0.81, CI = 0.39-1.72	N/A	[50]
	10	35						
Wang et al.	24	12	Multiple myeloma patients with COVID-19	Mount Sinai Hospital	Retrospective cohort	Statin use significantly associated with mortality. OR: 6.21, CI: 1.37-39.77, *p* = 0.012	N/A	[49]
	11	3						
Grasselli et al.	N/A	N/A	Patients admitted to ICUs in Lombardy with suspected SARS-CoV-2 infection	One of the network ICUs, Milan	Retrospective, observational study	Statins associated with higher mortality in univariate analysis. HR: 0.98, CI: 0.81-1.2, *p* = 0.87	Long-term treatment with statins, before ICU admission associated with higher mortality unadjusted analysis only. Multivariate analysis did not confirm association between any home therapies and increased mortality	[51]
	N/A	N/A						
Ayed et al.	10	93	Intensive care unit- (ICU-) admitted COVID-19 patients	Jaber Al-Ahmad Al Sabah Hospital, Kuwait	Retrospective cohort	OR: 0.49, CI: 0.11-2.08	N/A	[52]
	4	43						
Tan et al.	40	509	717 patients admitted	Tertiary center in Singapore for COVID-19 infection	Retrospective cohort	ATET Coeff: −0.04, CI: −0.16-0.08, *p* = 0.488	No significant differences in mortality	[36]
	2							
Peymani et al.	75	75	Hospitalized COVID-19 patients	Single tertiary hospital, Shiraz, Iran	Retrospective	HR: 0.76, CI: 0.16-3.72, *p* = 0.735	Nonsignificant association between statin use and reduction in mortality in patients with COVID-19	[47]
	N/A	N/A						
Nicholson et al.	511	531	1042 people with COVID-19 symptoms admitted	Mass General Brigham Hospitals	Retrospective cohort	OR: 0.50, CI: 0.27-0.93, *p* = 0.027	Chronic statin use associated with reduced in-hospital mortality	[53]
	N/A	N/A						
Lala et al.	984	1752	Hospitalized COVID-19-positive patients	1 of 5 Mount Sinai Health System hospitals in New York City	Multihospital retrospective cohort	HR: 0.57, CI: 0.47-0.69, *p* < 0.001	Statin use associated with improved survival	[54]
	N/A	N/A						
Krishnan et al.	81	71	Consecutive patients requiring mechanical ventilation from March 10 to April 15	St. Joseph Mercy Oakland Hospital	Retrospective observational	OR: 2.44, CI: 1.23-4.76, *p* = 0.0080	Statin use associated with increased mortality	[55]
	N/A	N/A						
Vahedian-Azimi et al.	326	525	Positive for SARS-CoV-2	Baqiyatallah University of Medical Sciences	Prospective observational	OR: 0.18, CI: 0.06–0.49 *p* = 0.0001	Statin use associated with decreased mortality	[10]
	8	282						
Butt et al.	843	3999	Danish citizens had a primary or secondary diagnosis code for COVID-19 infection	A Danish hospital, including inpatient, outpatient, and emergency department visits	Observational cohort study	HR 2.87 (95% CI 2.39 to 3.46)	Statin exposure was associated with a significantly higher risk of mortality compared with no statin exposure	[38]
	177 (21.0%)	311 (7.8%)						
Fan et al.	250	1897	Patients with COVID-19	Zhongnan Hospital of Wuhan University and Leishenshan Hospital in Wuhan, China	Retrospective study	Adjusted HR, 0.428; 95% CI, 0.169–0.907; *p* = 0.029	Statin use was associated with lower mortality	[39]
	6 (2.4%)	70 (3.7%)						
Israel et al.	N/A	N/A	Hospitalized COVID-19 patients were assigned to two distinct case-control cohorts. Control patients were taken from the general population	Clalit Health Services (CHS) data warehouse	Retrospective cohort	OR (95%CI) = 0.691 (0.444, 1.037), 0.072	Rosuvastatin has protective effects in this large population analysis	[56]
	N/A	N/A						
Israel et al.	N/A	N/A	Hospitalized COVID-19 patients were assigned to two distinct case-control cohorts. Case patients were nonhospitalized SARS-CoV-2-positive patients	Clalit Health Services (CHS) data warehouse	Case-control matched cohort	OR (95% CI) 0.530 (0.360, 0.766) *p* < 0.001	Rosuvastatin has protective effects in this large population analysis	[56]
	N/A	N/A						
Mughal et al.—abstract	44	76	Adult patients who were hospitalized with RT-PCR-confirmed SARS-CoV-2 infection	N/A	Retrospective cohort	N/A	N/A	[57]
	14 (31.8%)	7 (9.2%)						
Mallow et al.	5313	16363	COVID-19 patient	Database of inpatient and hospital-based outpatient detailed claims across more than 300 acute care hospitals in the US	Retrospective cohort	OR 0.54, 95% CI, 0.49–0.60; *p* < 0.001	Our findings suggest that patients administered statins in the hospital had a 46% lower risk of death than those not receiving statins	[28]
	N/A	N/A						
McCarthy et al.	107	140	Patients hospitalized with confirmed SARS-CoV-2 infection	Three Partners Healthcare hospitals (Massachusetts General Hospital, Brigham and Women's Hospital, and Newton-Wellesley Hospital)	Retrospective cohort study	Admitted to ICU or died OR: 1.18 (0.71-1.96)	N/A	[41]
	51	61						
Alamdari et al.	117	342	COVID-19 patients	Patients who were admitted to Shahid Modarres Hospital, which is a 279-bed tertiary referral center in Tehran, Iran	Retrospective cohort	OR: 0.27 (0.11–0.64)	Statin use history decreased the incidence of mortality dramatically	[58]
	6 (9.5%)	57 (16.7%)						
Soleimani et al.	66	188	Patients with COVID-19	Sina Hospital in Tehran, Iran	Retrospective observational study	OR: 0.93 (0.49–1.76)	N/A	[59]
	17 (25%)	51 (27%)						
Ayeh et al.	594	3853	Patients with a diagnosis of SARS-CoV-2 infection	Johns Hopkins Hospital and affiliated hospitals, Johns Hopkins Bayview Medical Center, Howard County General Hospital, Sibley Memorial Hospital, and Suburban Hospital, USA	Retrospective study	HR = 0.92, 95% CI (0.53–1.59)	The average treatment effect of statin use on COVID-19-related mortality in the matched groups was not statistically significant	[60]
	N/A	N/A						
Ahlström et al.	N/A	N/A	ICU COVID-19 patients	Sweden	Retrospective cohort study	OR = 0.72 (0.53-0.98) *p* = 0.034	Statins were protective of ICU death	[43]
	110	N/A						
An et al.	1074	9160	Patients diagnosed with COVID-19	South Korea	Nationwide cohort	OR: 4.11 (3.07-5.51)	N/A	[61]
	69 (6.4%)	159 (1.7%)						
Holman et al.	118995	142710	COVID-19 people with type 1 diabetes	The National Diabetes Audit (NDA), UK	Population-based cohort study	HR = 0.82 (0.65-1.03) *p* = 0.081	Association of prescription of statins with mortality in type 1 diabetes was not significant	[62]
	338	120						
Holman et al.	2099505	752245	COVID-19 people with type 2 diabetes	The National Diabetes Audit (NDA), UK	Population-based cohort study	HR = 0.72 (0.62-0.75) *p* < 0.001	In people with type 2 diabetes, prescription for statins was associated with reduced mortality	[62]
	7355	3086						
Inciardi et al.	25	74	Patients hospitalized for COVID-19 pneumonia	Civil Hospitals of Brescia, Lombardy, Italy	Retrospective cohort	OR = 1.89 (0.71-5.03)	N/A	[63]
	9 (36%)	17 (23%)						
Luo et al.	55	228	Patients with confirmed COVID-19	Tongji Hospital in Wuhan, China	Retrospective study	OR = 2.98 (0.65–13.76) *p* = 0.16	N/A	[27]
	N/A	N/A						
Ullah et al.	108	104	Confirmed COVID-19 patients	Primary, secondary, and tertiary electronic healthcare records (EHRs) of HPB patients in East London	Retrospective single-center cohort study	OR = 2.39 (1.25-4.56)	N/A	[64]
	36	18						
Ramachandran et al.	114	181	Patients admitted with a principal diagnosis of COVID-19	Tertiary care academic medical center in Brooklyn, New York	Retrospective cohort study	OR = 1.59 (0.84-3.02) *p* = 0.157	N/A	[65]
	N/A	N/A						
Izzi-Engbeaya et al.	N/A	N/A	Patients hospitalized with swab-positive COVID-19	ICHNT, which includes three hospitals admitting patients with COVID-19 (Charing Cross Hospital, Hammersmith Hospital, and St. Mary's Hospital), London	Retrospective cohort study	Primary outcome of death/ICU admission Estimate: −0.105 SE: 0.504 *p* = 0.835 OR = 1.49 (1.12-1.98)	N/A	[44]
	N/A	N/A						
Bifulco et al.	117	424	COVID-19 patients	Patients admitted to Humanitas Clinical and Research Hospital (Rozzano, Milan, Italy)	Retrospective cohort	Adjusted odds ratio (aOR): 0.75; 95% confidence interval (CI): 0.26–2.17; *p* = 0.593	Deaths were lower, although not significantly, in statin users with respect to nonstatin users	[66]
	N/A	N/A						
Oh et al.	N/A	N/A	Patients with COVID-19	NHIS-COVID-19 cohort database, South Korean	Retrospective cohort study	OR (95% CI) 0.74, (0.52, 1.05), *p* = 0.094	We found that it did not affect the hospital mortality of patients who were diagnosed with COVID-19	[29]
	N/A	N/A						
Maric et al.	2297	4594	COVID-19 patients	Cerner's large COVID-19 EHR database, USA	Retrospective cohort study	*p* = 0.0183	We observed a small, but statistically significant, decrease in mortality among patients prescribed statins (16.1%) when compared with matched COVID-19-positive controls (18.0 to 20.6%)	[67]
	369 (16.1%)	845 (18.39%)						
Mitacchione et al.	179	663	Patients hospitalized for COVID-19	Hospitals include Luigi Sacco Hospital, Milan; Policlinico Umberto I Hospital, Rome; Spedali Civili Hospital, Brescia; and Humanitas Gavazzeni Hospital, Bergamo, Italia	Observational multicenter study	*p* = 0.006	Statin users appeared to show higher mortality rates	[42]
	52 (%29)	130 (%20)						

N/A: not available.

## Data Availability

No original (raw) data was produced for this systematic review.

## Supplementary Materials

Supplementary Materials Supplementary Table 1: quality assessment of studies by *Newcastle-Ottawa* scale (NOS). Supplementary Figure 1: flow chart of the study selection process.
