# Supplementary material for: Improved COVID-19 Outcomes following Statin Therapy: An Updated Systematic Review and Meta-analysis
Source: Biomed Res Int. 2021 Sep 23;2021:1901772. doi: 10.1155/2021/1901772 (PMC8463212; doi:10.1155/2021/1901772)
Supplement: Supplementary Materials — Supplementary Table 1: quality assessment of studies by Newcastle-Ottawa scale (NOS). Supplementary Figure 1: flow chart of the study selection process. [file 1901772.f1.doc]

**
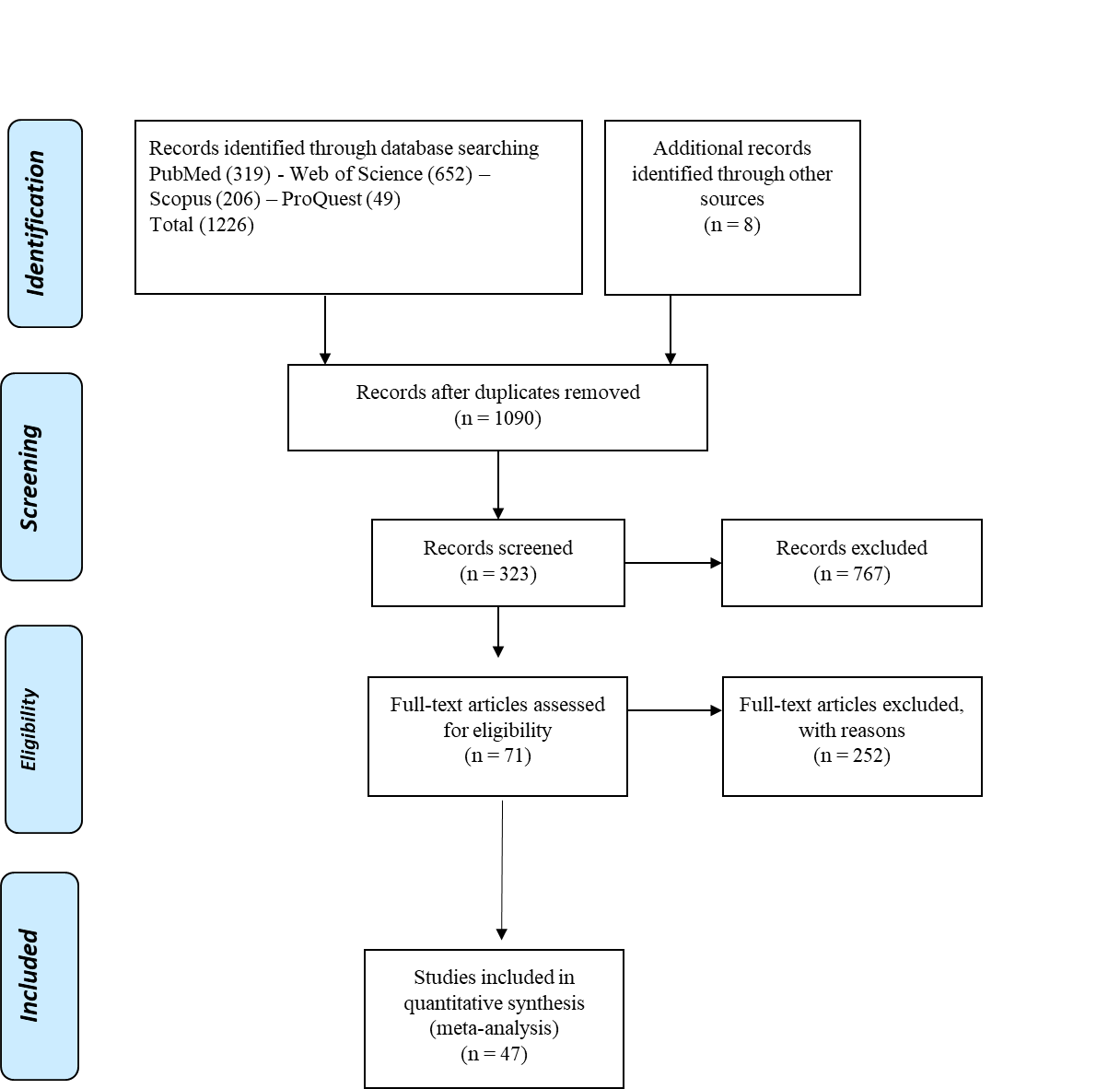
**

**Supplementary Figure 1:** Flow chart of the study selection process.

**Supplementary Table 1:** Quality assessment of studies by Newcastle*-*Ottawa scale (NOS)

| **Studies** | **Selection of cohorts** | **Comparability of cohorts** | **Outcome** | **Total Score** | **Quality** |
| --- | --- | --- | --- | --- | --- |
| Gupta et al. [1] | ★★★ | ★★ | ★★ | 7 | Good |
| Masana et al. [2] | ★★★ | ★ | ★★ | 6 | Good |
| Zhang et al. [3] | ★★★★ | ★★ | ★★★ | 9 | Good |
| Rossi et al. [4] | ★★★ | ★ | ★★ | 6 | Good |
| Cariou et al. [5] | ★★★ | ★★ | ★★★ | 8 | Good |
| Saeed et al. [6] | ★★★★ | ★★ | ★★★ | 9 | Good |
| Song et al. [7] | ★★★★ | ★★ | ★★ | 8 | Good |
| De Spiegeleer et al. [8] | ★★★ | ★★ | ★★ | 7 | Good |
| Rodriguez-Nava et al. [9] | ★★★ | ★ | ★★ | 6 | Good |
| Zenga et al. [10] | ★★★ | ★★ | ★★ | 7 | Good |
| Nguyen et al. [11] | ★★★★ | ★★ | ★★★ | 8 | Good |
| Wang et al. [10] | ★★★ | ★ | ★★ | 6 | Good |
| Grasselli et al. [12] | ★★★★ | ★★ | ★★★ | 9 | Good |
| Yan et al. [13] | ★★★ | ★★ | ★★ | 7 | Good |
| Ayed et al. [14] | ★★★★ | ★★ | ★★ | 8 | Good |
| Tan et al. [15] | ★★★ | ★★ | ★★ | 7 | Good |
| Peymani et al. [16] | ★★★ | ★★ | ★★ | 7 | Good |
| Nicholson et al. [17] | ★★★★ | ★★ | ★★★ | 9 | Good |
| Lala et al. [18] | ★★★★ | ★★ | ★★★ | 9 | Good |
| Krishnan et al. [19] | ★★★ | ★★ | ★★ | 7 | Good |
| Argenziano et al. [20] | ★★★★ | ★★ | ★★★ | 9 | Good |
| Daniels et al. [21] | ★★★★ | ★★ | ★★★ | 9 | Good |
| Dreher et al. [22] | ★★★★ | ★★ | ★★ | 8 | Good |
| Vahedian-Azimi et al. [23] | ★★★★ | ★★ | ★★★ | 9 | Good |
| Butt et al.[24] | ★★★★ | ★★ | ★★ | 8 | Good |
| Fan et al.[25] | ★★★★ | ★★ | ★★★ | 9 | Good |
| Israel et al.[26] | ★★★★ | ★★ | ★★ | 8 | Good |
| Bifulco et al.[27] | ★★★ | ★ | ★★ | 6 | Good |
| Mallow et al.[28] | ★★★ | ★★ | ★★★ | 8 | Good |
| Alamdari et al.[29] | ★★★ | ★ | ★★ | 6 | Good |
| Soleimani et al.[30] | ★★★★ | ★ | ★★ | 7 | Good |
| Oh et al.[31] | ★★★ | ★★ | ★★ | 7 | Good |
| Maric et al.[32] | ★★★ | ★★ | ★★★ | 8 | Good |
| Mitacchione et al.[33] | ★★★★ | ★★ | ★★ | 8 | Good |
| Ayeh et al.[34] | ★★★ | ★★ | ★★ | 7 | Good |
| An et al.[25] | ★★★★ | ★★ | ★★ | 8 | Good |
| Holman et al.[35] | ★★★★ | ★★ | ★★★ | 9 | Good |
| Inciardi et al.[36] | ★★★ | ★★ | ★★ | 7 | Good |
| Luo et al.[37] | ★★★ | ★★ | ★★ | 7 | Good |
| Ullah et al.[38] | ★★★★ | ★★ | ★★ | 8 | Good |
| Ramachandran et al.[39] | ★★★ | ★★ | ★★★ | 8 | Good |
| Izzi-Engbeaya et al.(58) | ★★★ | ★★ | ★★ | 7 | Good |
| McCarthy el.[40] | ★★★★ | ★★ | ★★★ | 9 | Good |

Thresholds for converting the Newcastle-Ottawa scales to AHRQ standards (good, fair, and poor):

Good quality: 3 or 4 stars in selection domain AND 1 or 2 stars in comparability domain AND 2 or 3 stars in outcome/exposure domain

Fair quality: 2 stars in selection domain AND 1 or 2 stars in comparability domain AND 2 or 3 stars in outcome/exposure domain

Poor quality: 0 or 1 star in selection domain OR 0 stars in comparability domain OR 0 or 1 stars in outcome/exposure domain

[1] Gupta A, Madhavan MV, Poterucha TJ, DeFilippis EM, Hennessey JA, Redfors B, et al.,“Association between antecedent statin use and decreased mortality in hospitalized patients with COVID-19,“, 2020.

[2] Masana L, Correig E, Rodríguez-Borjabad C, Anoro E, Arroyo JA, Jericó C, et al.,“EFFECT oF STATIN THERAPY oN SARS-CoV-2 INFECTION-RELATED,“ *European Heart Journal-Cardiovascular Pharmacotherapy*, 2020.

[3] Zhang X-J, Qin J-J, Cheng X, Shen L, Zhao Y-C, Yuan Y, et al.,“In-hospital use of statins is associated with a reduced risk of mortality among individuals with COVID-19,“ *Cell metabolism*, 2020;32(2):176-87. e4.

[4] Rossi R, Talarico M, Coppi F, Boriani G,“Protective role of statins in COVID 19 patients: importance of pharmacokinetic characteristics rather than intensity of action,“ *Internal and Emergency Medicine*, 2020:1-4.

[5] Cariou B, Goronflot T, Rimbert A, Boullu S, Le May C, Moulin P, et al.,“Routine use of statins and increased mortality related to COVID-19 in inpatients with type 2 diabetes: Results from the CORONADO study,“ *Diabetes Metab*, 2020.

[6] Saeed O, Castagna F, Agalliu I, Xue X, Patel SR, Rochlani Y, et al.,“Statin Use and In‐Hospital Mortality in Diabetics with COVID‐19,“ *Journal of the American Heart Association*, 2020:e018475.

[7] Song SL, Hays SB, Panton CE, Mylona EK, Kalligeros M, Shehadeh F, et al.,“Statin Use Is Associated with Decreased Risk of Invasive Mechanical Ventilation in COVID-19 Patients: A Preliminary Study,“ *Pathogens*, 2020;9(9):759.

[8] De Spiegeleer A, Bronselaer A, Teo JT, Byttebier G, De Tre G, Belmans L, et al.,“The effects of ARBs, ACEIs and statins on clinical outcomes of COVID-19 infection among nursing home residents,“ *medRxiv*, 2020.

[9] Rodriguez-Nava G, Trelles-Garcia DP, Yanez-Bello MA, Chung CW, Trelles-Garcia VP, Friedman HJ,“Atorvastatin associated with decreased hazard for death in COVID-19 patients admitted to an ICU: a retrospective cohort study,“ *Critical Care*, 2020;24(1):1-2.

[10] Zenga H, Zhang T, He X, Du Y, Tong Y, Wang X, et al.,“Impact of Hypertension on Progression and Prognosis in Patients with COVID-19,“, 2020.

[11] Nguyen AB, Upadhyay GA, Chung B, Smith B, Besser SA, Johnson JA, et al.,“Outcomes and Cardiovascular Comorbidities in a Predominantly African-American Population with COVID-19,“ *medRxiv*, 2020.

[12] Grasselli G, Greco M, Zanella A, Albano G, Antonelli M, Bellani G, et al.,“Risk factors associated with mortality among patients with COVID-19 in intensive care units in Lombardy, Italy,“ *JAMA internal medicine*, 2020;180(10):1345-55.

[13] Yan H, Valdes AM, Vijay A, Wang S, Liang L, Yang S, et al.,“Role of Drugs Affecting the Renin-Angiotensin-Aldosterone System on Susceptibility and Severity of COVID-19: A Large Case-Control Study from Zheijang Province, China,“ *medRxiv*, 2020.

[14] Ayed M, Borahmah AA, Yazdani A, Sultan A, Mossad A, Rawdhan H,“Assessment of clinical characteristics and mortality-associated factors in COVID-19 Critical cases in Kuwait,“ *Medical Principles and Practice*, 2020.

[15] Tan WY, Young BE, Lye DC, Chew DE, Dalan R,“Statin use is associated with lower disease severity in COVID-19 infection,“ *Scientific Reports*, 2020;10(1):1-7.

[16] Peymani P, Dehesh T, Aligolighasemabadi F, Sadeghdoust M, Kotfis K, Ahmadi M, et al.,“Statins in Patients with COVID-19: A Retrospective Cohort Study in Iranian COVID-19 Patients,“, 2020.

[17] Nicholson CJ, Wooster L, Sigurslid HH, Li RF, Jiang W, Tian W, et al.,“Estimating Risk of Mechanical Ventilation and Mortality Among Adult COVID-19 patients Admitted to Mass General Brigham: The VICE and DICE Scores,“ *medRxiv*, 2020.

[18] Lala A, Johnson KW, Januzzi JL, Russak AJ, Paranjpe I, Richter F, et al.,“Prevalence and impact of myocardial injury in patients hospitalized with COVID-19 infection,“ *Journal of the American College of Cardiology*, 2020;76(5):533-46.

[19] Krishnan S, Patel K, Desai R, Sule A, Paik P, Miller A, et al.,“Clinical comorbidities, characteristics, and outcomes of mechanically ventilated patients in the State of Michigan with SARS-CoV-2 pneumonia,“ *Journal of clinical anesthesia*, 2020;67:110005.

[20] Argenziano MG, Bruce SL, Slater CL, Tiao JR, Baldwin MR, Barr RG, et al.,“Characterization and clinical course of 1000 patients with coronavirus disease 2019 in New York: retrospective case series,“ *bmj*, 2020;369.

[21] Daniels LB, Sitapati AM, Zhang J, Zou J, Bui QM, Ren J, et al.,“Relation of statin use prior to admission to severity and recovery among COVID-19 inpatients,“ *The American journal of cardiology*, 2020.

[22] Dreher M, Kersten A, Bickenbach J, Balfanz P, Hartmann B, Cornelissen C, et al.,“The characteristics of 50 hospitalized COVID-19 patients with and without ARDS,“ *Deutsches Ärzteblatt International*, 2020;117(10):271.

[23] Vahedian-Azimi A, Rahimibashar F, Najafi A, Kidde J, Shahriary A, Shojaei S, et al.,“Association of in-hospital use of statins, aspirin and renin-angiotensin-aldosterone inhibitors with mortality and ICU admission due to COVID-19,“ *Unpublished*, 2020.

[24] Butt JH, Gerds TA, Schou M, Kragholm K, Phelps M, Havers-Borgersen E, et al.,“Association between statin use and outcomes in patients with coronavirus disease 2019 (COVID-19): a nationwide cohort study,“ *Bmj Open*, 2020;10(12).

[25] Fan Y, Guo T, Yan F, Gong M, Zhang XA, Li C, et al.,“Association of Statin Use With the In-Hospital Outcomes of 2019-Coronavirus Disease Patients: A Retrospective Study,“ *Frontiers in medicine*, 2020;7:584870.

[26] Israel A, Schaffer A, Cicurel A, Feldhamer I, Tal A, Cheng K, et al.,“Large population study identifies drugs associated with reduced COVID-19 severity,“ *medRxiv*, 2020.

[27] Bifulco M, Ciccarelli M, Bruzzese D, Dipasquale A, Lania AG, Mazziotti G, et al.,“The benefit of statins in SARS-CoV-2 patients: further metabolic and prospective clinical studies are needed,“ *Endocrine*, 2021;71(2):270-2.

[28] Mallow PJ, Belk KW, Topmiller M, Hooker EA,“Outcomes of Hospitalized COVID-19 Patients by Risk Factors: Results from a United States Hospital Claims Database,“ *J Health Econ Outcomes Res*, 2020;7(2):165-74.

[29] Alamdari NM, Afaghi S, Rahimi FS, Tarki FE, Tavana S, Zali A, et al.,“Mortality Risk Factors among Hospitalized COVID-19 Patients in a Major Referral Center in Iran,“ *Tohoku J Exp Med*, 2020;252(1):73-84.

[30] Soleimani A, Kazemian S, Karbalai Saleh S, Aminorroaya A, Shajari Z, Hadadi A, et al.,“Effects of Angiotensin Receptor Blockers (ARBs) on In-Hospital Outcomes of Patients With Hypertension and Confirmed or Clinically Suspected COVID-19,“ *Am J Hypertens*, 2020;33(12):1102-11.

[31] Oh TK, Song I-A, Jeon Y-T,“Statin Therapy and the Risk of COVID-19: A Cohort Study of the National Health Insurance Service in South Korea,“ *J Pers Med*, 2021;11(2):116.

[32] Marić I, Oskotsky T, Kosti I, Le B, Wong RJ, Shaw GM, et al.,“Decreased Mortality Rate Among COVID-19 Patients Prescribed Statins: Data From Electronic Health Records in the US,“ *Frontiers in medicine*, 2021;8:639804.

[33] Mitacchione G, Schiavone M, Curnis A, Arca M, Antinori S, Gasperetti A, et al.,“Impact of prior statin use on clinical outcomes in COVID-19 patients: data from tertiary referral hospitals during COVID-19 pandemic in Italy,“ *Journal of clinical lipidology*, 2021;15(1):68-78.

[34] Ayeh S, Abbey E, Khalifa B, Nudotor R, Osei A, Chidambaram V, et al. Statin Use Is Associated with More Severe COVID-19 Disease in Hospitalized Patients. SSRN; 2021.

[35] Holman N, Knighton P, Kar P, O'Keefe J, Curley M, Weaver A, et al.,“Risk factors for COVID-19-related mortality in people with type 1 and type 2 diabetes in England: a population-based cohort study,“ *Lancet Diabetes Endocrinol*, 2020;8(10):823-33.

[36] Inciardi RM, Adamo M, Lupi L, Cani DS, Di Pasquale M, Tomasoni D, et al.,“Characteristics and outcomes of patients hospitalized for COVID-19 and cardiac disease in Northern Italy,“ *Eur Heart J*, 2020;41(19):1821-9.

[37] Luo P, Qiu L, Liu Y, Liu X-L, Zheng J-L, Xue H-Y, et al.,“Metformin Treatment Was Associated with Decreased Mortality in COVID-19 Patients with Diabetes in a Retrospective Analysis,“ *Am J Trop Med Hyg*, 2020;103(1):69-72.

[38] Ullah AZMD, Sivapalan L, Chelala C, Kocher HM,“COVID-19 in patients with hepatobiliary and pancreatic diseases in East London: A single-centre cohort study,“ *medRxiv*, 2020:2020.09.07.20189621.

[39] Ramachandran P, Perisetti A, Gajendran M, Jean-Louis F, Bansal P, Dwivedi AK, et al.,“Pre-hospitalization proton pump inhibitor use and clinical outcomes in COVID-19,“ *European Journal of Gastroenterology & Hepatology*, 9000;Publish Ahead of Print.

[40] McCarthy CP, Murphy S, Jones-O'Connor M, Olshan DS, Khambhati JR, Rehman S, et al.,“Early clinical and sociodemographic experience with patients hospitalized with COVID-19 at a large American healthcare system,“ *EClinicalMedicine*, 2020;26:100504.
